# The Diagnostic Utility of the Basal Luteinizing Hormone Level and Single 60-Minute Post GnRH Agonist Stimulation Test for Idiopathic Central Precocious Puberty in Girls

**DOI:** 10.3389/fendo.2021.713880

**Published:** 2021-08-12

**Authors:** Ruixue Cao, Jinrong Liu, Pinguo Fu, Yonghai Zhou, Zhe Li, Peining Liu

**Affiliations:** ^1^Department of Pediatrics, The Second Affiliated Hospital and Yuying Children’s Hospital of Wenzhou Medical University, Wenzhou, China; ^2^Department of Anesthesiology, The Second Affiliated Hospital and Yuying Children’s Hospital of Wenzhou Medical University, Wenzhou, China

**Keywords:** central precocious puberty, GnRH agonist stimulation test, luteinizing hormone, follicle-stimulating hormone, pediatrics

## Abstract

**Objective:**

The present study aimed to assess the diagnostic utility of the Luteinizing hormone (LH) levels and single 60-minute post gonadotropin-releasing hormone (GnRH) agonist stimulation test for idiopathic central precocious puberty (CPP) in girls.

**Methods:**

Data from 1,492 girls diagnosed with precocious puberty who underwent GnRH agonist stimulation testing between January 1, 2016, and October 8, 2020, were retrospectively reviewed. LH levels and LH/follicle-stimulating hormone (FSH) ratios were measured by immuno-chemiluminescence assay before and at several timepoints after GnRH analogue stimulation testing. Mann–Whitney U test, Spearman’s correlation, χ^2^ test, and receiver operating characteristic (ROC) analyses were performed to determine the diagnostic utility of these hormone levels.

**Results:**

The 1,492 subjects were split into two groups: an idiopathic CPP group (*n* = 518) and a non-CPP group (*n* = 974). Basal LH levels and LH/FSH ratios were significantly different between the two groups at 30, 60, 90, and 120 minutes after GnRH analogue stimulation testing. Spearman’s correlation analysis showed the strongest correlation between peak LH and LH levels at 60 minutes after GnRH agonist stimulation (*r* = 0.986, *P* < 0.001). ROC curve analysis revealed that the 60-minute LH/FSH ratio yielded the highest consistency, with an area under the ROC curve (AUC) of 0.988 (95% confidence interval [CI], 0.982–0.993) and a cut-off point of 0.603 mIU/L (sensitivity 97.3%, specificity 93.0%). The cut-off points of basal LH and LH/FSH were 0.255 mIU/L (sensitivity 68.9%, specificity 86.0%) and 0.07 (sensitivity 73.2%, specificity 89.5%), respectively, with AUCs of 0.823 (95% CI, 0.799–0.847) and 0.843 (95% CI, 0.819–0.867), respectively.

**Conclusions:**

A basal LH value greater than 0.535 mIU/L can be used to diagnose CPP without a GnRH agonist stimulation test. A single 60-minute post-stimulus gonadotropin result of LH and LH/FSH can be used instead of a GnRH agonist stimulation test, or samples can be taken only at 0, 30, and 60 minutes after a GnRH agonist stimulation test. This reduces the number of blood draws required compared with the traditional stimulation test, while still achieving a high level of diagnostic accuracy.

## Introduction

Female precocious puberty is generally divided into three types based on whether the hypothalamic–pituitary–gonadal (HPG) axis function is activated in advance: central precocious puberty (CPP), peripheral precocious puberty (PPP), and incomplete precocious puberty (IPP). CPP is a disease of the endocrine system caused by premature activation of the HPG axis. It can lead to a variety of clinical manifestations, including accelerated growth velocity, progressive pubertal development, and advanced bone age (BA). CPP can be divided into types according to its etiology: secondary CPP and idiopathic CPP (ICPP). The former is secondary to organic diseases of the central nervous system, such as a thalamus or pituitary tumor; the latter occurs without definite organic lesions (1).

The gonadotropin-releasing hormone (GnRH)-stimulation test is considered the gold standard for the diagnosis of CPP in subjects with early symptoms of puberty (2). However, the test requires multiple blood samples to be taken to identify the peak levels of luteinizing hormone (LH) and follicle-stimulating hormone (FSH), which can be inconvenient and often increases fear in pediatric patients. Many recent studies have investigated whether baseline LH or a single post-stimulation LH value is adequate for the diagnosis of CPP (3–9). However, these reports often only evaluated one index in a small sample, which reduces the credibility of their findings.

The present study examined the utility of basal LH and LH/FSH ratio with GnRH agonist stimulation testing at different times (30, 60, 90, and 120 minutes after stimulus) for diagnosing idiopathic CPP in 1,492 subjects in order to identify the most feasible and effective diagnostic index to be collected at a single time point.

## Materials and Methods

### Subjects

The data of all female patients diagnosed with precocious puberty between January 1, 2016, and October 8, 2020, in the Second Affiliated Hospital and Yuying Children’s Hospital of Wenzhou Medical University were retrospectively reviewed. Only those who met all the inclusion criteria were selected for study (*n* = 1,492).

Inclusion criteria (based on a consensus statement) (10): (1) secondary sexual development before the age of 8, (2) increased ovarian and uterine size with several follicles > 4 mm in diameter on pelvic ultrasound; (3) brain magnetic resonance imaging was performed; (4) GnRH agonist stimulation testing was performed.

Exclusion criterion: (1) demonstrable organic diseases that could affect HPG axis function, including a thalamus, pituitary tumor, other central nervous system diseases, and an ovarian cyst.

### GnRH Agonist Stimulation Test

The serum concentrations of LH and FSH were measured by immuno-chemiluminescence assay, with fasting serum samples collected at 9.00 a.m. (LH sensitivity = 0.01 IU/L; FSH sensitivity = 0.05 IU/L). Next, triptorelin acetate (Ferring AG, Saint-Prex, Switzerland) was subcutaneously injected, with a dosage of 2.5 ug/kg (maximum dosage = 100 ug). Blood samples were collected 30, 60, 90, and 120 minutes later and assayed for LH and FSH. Patients with a peak LH value > 5.0 mIU/L and peak LH/FSH ratio > 0.6 were defined as having ICPP and placed in the ICPP group. Those who did not meet either criterion were placed in the non-CPP group and used as a control group.

### Statistical Analysis

Data were analyzed using SPSS 24.0 software (IBM Corp., Armonk, NY, USA). A Mann–Whitney U test was performed to compare clinical parameters between groups, and the results were given as median and range. Patients of the same age and sex, with a body mass index (BMI) higher than 95%, were also diagnosed with obesity. The prevalence of obesity in both groups was analyzed using a χ^2^ test.

Spearman’s correlation analysis was undertaken to identify and evaluate the correlation between peak LH and LH levels at 0, 60, 90, and 120 minutes after GnRH agonist stimulation.

Receiver operating characteristic (ROC) analysis was performed to evaluate the sensitivity and specificity of estradiol, basal LH, LH/FSH ratio, and single results for 30, 60, 90, and 120 minutes after GnRH agonist stimulation. The Youden index (sensitivity + specificity–1) was calculated to evaluate the ROC curve cut-off point for each assay. Differences were considered statistically significant at *P* < 0.05.

## Results

All 1578 cases of female patients diagnosed with precocious puberty during a five-year period were reviewed. All of them had the development of secondary sexual characteristics such as an increase in growth velocity, development of breast or pubic hair before 8 years old. These secondary sexual characteristics should appear for more than 3 months, and Tanner stages were at least above grade 2 with several follicles > 4 mm in diameter showed by pelvic ultrasound. Among of them, 75 patients were excluded because of refusing the test of GnRH agonist stimulation, 8 patients were excluded because of having organic diseases, and 3 patients were excluded because of both having organic diseases and refusing the test of GnRH agonist stimulation. Thus, a total of 86 patients were excluded. Finally, 1,492 cases were included in the present study (*n* = 518 ICPP group; *n* = 974 non-CPP group). In ICPP group, the case numbers in Tanner stage 2, 3, 4 were 222 (42.86%), 268 (51.74%), 28 (5.41%). In non-CPP group, the numbers were 643 (66.02%), 317 (32.55%), 14(1.44%), respectively. Their other clinical and demographic characteristics are shown in **Table 1**. The patients in the ICPP group were found to be older than those in the non-CPP group and had higher values for height, weight, BMI, BA, and BA-CA. In the ICPP group, 49 cases were complicated with obesity, compared with 98 cases in the non-CPP group. There was no significant difference in the proportion of obesity between the two groups (χ^2^ = 0.138, *P* = 0.710).

**Table 1:** Subjects’ clinical and laboratory characteristics.

	CPP (n = 518)	Non-CPP (n = 974)	P-value
Age (years)	7.97 (0.60)	7.46 (0.83)	<0.001
BA (years)	9.93 (1.17)	8.88 (1.42)	<0.001
BA-CA (years)	2.01 (1.64)	1.40 (1.10)	<0.001
Height	133.12 (5.88)	129.16 (6.94)	<0.001
Height-SDS	1.00 (0.90)	0.85 (0.94)	0.002
weight	30.22 (4.87)	27.95 (5.46)	<0.001
Weight-SDS	0.91 (0.87)	0.75 (0.99)	0.004
BMI	17.01 (2.14)	16.64 (2.33)	<0.001
BMI-SDS	0.5 (0.96)	0.38 (1.14)	0.041
estradiol	39.36 (22.36)	28.90 (14.75)	<0.001
LH0	0.88 (1.32)	0.12 (0.46)	<0.001
LH30	19.28 (14.68)	4.40 (2.95)	<0.001
LH60	20.80 (15.96)	4.79 (3.33)	<0.001
LH90	19.74 (14.28)	4.76 (3.13)	<0.001
LH120	18.92 (6.82)	4.81 (3.00)	<0.001
LH-P	22.73 (16.08)	5.26 (3.40)	<0.001
FSH0	4.03 (2.11)	3.12 (1.95)	<0.001
FSH30	12.23 (4.73)	11.36 (4.37)	0.001
FSH60	15.28 (6.39)	14.50 (5.83)	0.056
FSH90	17.01 (6.87)	16.79 (6.63)	0.850
FSH120	18.29 (6.82)	18.95 (7.28)	0.077
FSH-P	18.56 (6.98)	19.11 (7.26)	0.992
LH/FSH0	0.18 (0.24)	0.23 (0.50)	<0.001
LH/FSH30	1.52 (0.96)	0.39 (0.20)	<0.001
LH/FSH60	1.30 (0.69)	0.33 (0.18)	<0.001
LH/FSH90	1.13 (0.57)	0.29 (0.15)	<0.001
LH/FSH120	1.03 (0.52)	0.27 (0.16)	<0.001
LH/FSH-P	1.20 (0.60)	0.28 (0.15)	<0.001

LH, FSH, and LH/FSH levels at 0, 30, 60, 90, and 120 minutes post stimulus gonadotropin are expressed as LH0, LH30, LH60, LH90, and LH120; FSH0, FSH30, FSH60, FSH90, and FSH120; and LH/FSH0, LH/FSH30, LH/FSH60, LH/FSH90, and LH/FSH120, respectively. The peak level of LH，FSH，LH/FSH are expressed as LH-P，FSH-P，LH/FSH-P, respectively.

LH levels and LH/FSH ratios were found to be significantly greater in the ICPP group than in the non-CPP group at 0, 30, 60, 90, and 120 minutes after stimulation (all *P* < 0.05). With regard to FSH, only the baseline value and FSH30 were significantly higher in the ICPP group than in the non-CPP group (*P* < 0.05).

**Figure 1:**
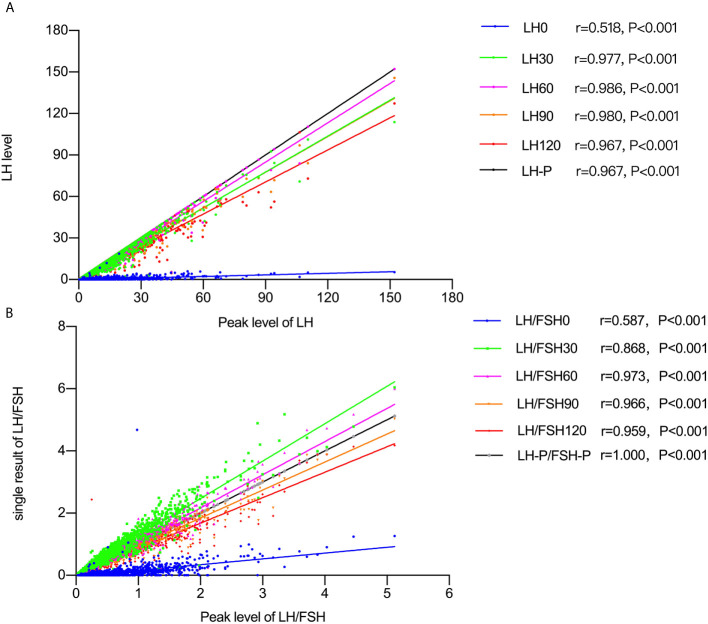
**(A)** Spearman’s correlation results between peak LH and the level at 0, 60, 90, and 120 minutes after GnRH agonist stimulation. **(B)** Spearman’s correlation results between peak LH/FSH ratio levels and the level at 0, 60, 90, and 120 minutes after GnRH agonist stimulation.

Spearman’s correlation analysis (**Figure 1**) showed positive correlations between peak LH values and LH0, LH30, LH60, LH90, and LH120, with the strongest correlation for LH60 (*r* = 0.986, *P* < 0.001). The LH/FSH ratios measured at different times after GnRH agonist stimulation were also compared, with LH/FSH60 showing the highest correlation (*r* = 0.973, *P* < 0.001).

**Figure 2:**
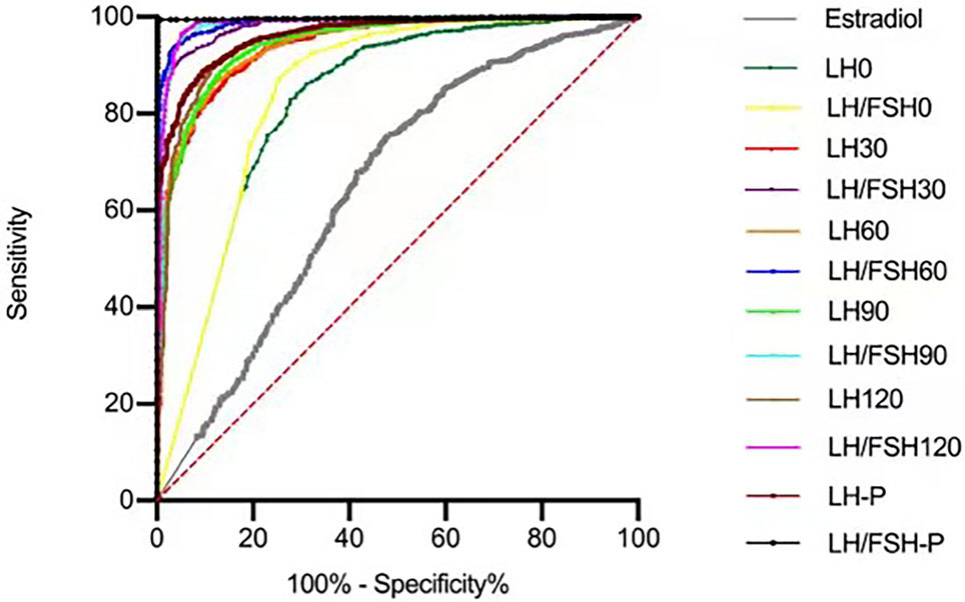
ROC curves used to evaluate the sensitivity and specificity at each level for LH and LH/FSH post GnRH agonist stimulus.

ROC curves were generated, and the optimal cut-off values for distinguishing precocious puberty from non-CPP were determined for each hormone based on the sensitivity, specificity, and area under the curve (AUC) (**Table 2**, **Figure 2**). The highest AUC was for LH/FSH60 (0.988), with a cut-off value of 0.603 (sensitivity 97.3%, specificity 93.0%), followed by a stimulated LH60 > 7.65m UI/l (sensitivity 87.6%, specificity 86.6%).

**Table 2:** Gonadotropin cut point for best discriminating pubertal status from ROC curves.

	AUC	95%CI		Cut point	Sensitivity	Specificity	Youden’s J index
Estradiol	0.650	0.619	0.680	32.85	0.525	0.749	0.274
LH0	0.823	0.799	0.847	0.255	0.689	0.86	0.549
FSH0	0.632	0.603	0.662	3.347	0.568	0.629	0.197
LH/FSH0	0.843	0.819	0.867	0.070	0.732	0.895	0.627
LH30	0.941	0.929	0.953	7.345	0.855	0.877	0.732
LH/FSH30	0.979	0.971	0.986	0.682	0.950	0.916	0.866
LH60	0.945	0.934	0.956	7.650	0.876	0.866	0.742
LH/FSH60	0.988	0.982	0.993	0.603	0.973	0.930	0.903
LH90	0.945	0.934	0.956	8.175	0.869	0.888	0.757
LH/FSH90	0.985	0.978	0.992	0.596	0.936	0.974	0.910
LH120	0.951	0.939	0.962	8.825	0.865	0.916	0.781
LH/FSH120	0.985	0.978	0.992	0.540	0.948	0.969	0.917
LH-P	0.964	0.956	0.972	8.955	0.903	0.889	0.792
LH/FSH-P	0.997	0.994	1.000	0.601	0.998	0.995	0.993

## Discussion

Precocious puberty refers to the development of secondary sex characteristics before the age of 8 in girls and 9 in boys. However, the precise timing of when secondary signs begin is still controversial, partly because of differences between racial groups. The lower age at onset for breast development reported in some studies also highlights the challenges in current diagnostic decision making for CPP (11–13).

In the present study, age, BA, BA-CA, height, weight, BMI, and estradiol levels were significantly different between the two groups. The average age in the ICPP group was 7.57 (0.60), which was 0.48 years older than the average age of the non-CPP group. The height-SDS was different between the two groups (P=0.002), which indicated that the growth rate in ICPP was higher than it in non-CPP. However, both patients with ICPP and non-CPP showed an increase in growth velocity, which is not helpful to distinguish ICPP from non-CPP.

Since early puberty exposure to sex hormones accelerates bone maturation, BA assessment is part of the initial examination when CPP is suspected based on early symptoms of adolescence. The BA of rapid PPP is usually > 2 SD, but in adrenal precocious puberty or precocious puberty it is usually only 1–2 SD higher than chronological age (14). However, it may be lower if CPP is diagnosed early. In some children, CPP progression may be slow and bone maturation rates may be near normal (15), so the BA level might not be related to the age of pubertal onset (16). In the present study, BA was more advanced in patients in the ICPP group than in the non-CPP group, but it was not helpful for distinguishing between the two groups. This result is consistent with previous studies. Similarly, estradiol was significantly higher in the ICPP group than in the non-CPP group but was not helpful in distinguishing between the groups.

Although the present study found a higher BMI in the ICPP group than in the non-CPP group, there was no significant difference between the two groups in the proportion of obese patients. The relationship between obesity and puberty remains controversial, although a growing body of evidence shows a connection between the two (17–19). The relationship between obesity and precocity may be mediated through leptin and its interaction with kisspeptin signaling, which is an important regulatory system in adolescence (20). The peripheral effects of obesity may also promote precocity: aromatase produced by adipose tissue can increase the conversion of androgen into estrogen (21), and obese children have higher estradiol concentrations than their non-obese peers (22). Other factors, such as nutrition, epigenetics, or endocrine-disrupting chemicals, may also link the onset of puberty and obesity (21).

Attempts have been made to find a more acceptable and widely available alternative to the GnRH-stimulation test. Some researchers have proposed LH > 0.2–0.3 mIU/L from a random blood sample as a reliable screening cut-off for CPP (6, 23, 24). The present study also assessed the diagnostic utility of basal LH levels and found that the cut-off point was 0.255 mIU/L (sensitivity 68.9%, specificity 86.0%), which is similar to the cut-off points found in previous reports (1, 9). In the present study, the baseline LH/FSH ratio had a cut-off point of 0.07, with a relatively low sensitivity and specificity of 73.2% and 89.5%, respectively. However, when the LH value was 0.535 mIU/L, the specificity reached 94.9% and the sensitivity was 50.0%. Therefore, when the baseline value of LH is > 0.535 mIU/L, a diagnosis of CPP can be confirmed without a GnRH agonist stimulation test. These patients who have a basal LH level well within the pubertal range can be considered to have CPP if the clinical findings are consistent. When the basal LH level is between 0.255 and 0.535 mIU/L or the LH/FSH level is > 0.07, clinical manifestations and BA results should be evaluated carefully, but most cases can be confirmed without the GnRH agonist stimulation test. If the LH is < 0.255 mIU and LH/FSH0 is < 0.07, GnRH stimulation should be performed.

A previous study reported that a peak stimulated LH value > 4–6 mIU/L and an LH/FSH ratio > 0.66 is the diagnostic standard of CPP (25). However, this test is inconvenient for pediatric patients because it is time consuming and requires multiple sampling points. To simplify diagnosis, the present study evaluated the consistency between measurement at a single time point and the traditional stimulation test. The results showed that LH level and the LH/FSH ratio taken at any of the four timepoints (30, 60, 90, and 120 minutes) had fairly high consistency with the peak LH level and LH/FSH ratio. Further analysis showed that the 60-minute results had the highest consistency with the peak LH and LH/FSH ratio values.

The pituitary response to GnRH occurs as two pools, an initial, acutely releasable pool, and a slower response in the form of a reserve pool. The level of LH value increased rapidly to a relatively stable level or actually decreased within 30 to 60 minutes after the infusion, and then increased slowly again within 90-120 minutes until the infusion stopped at 4 hours (26). However, our objective is not to find the peak of LH, but to find a simple but effective diagnostic methods to distinguish CPP, which is more acceptable for patients. The cut-off points of LH60 and LH/FSH60 were 7.65 mIU/L (sensitivity 87.6% and specificity 86.6%) and 0.603 mIU/L (sensitivity 97.3%, specificity 93.0%), respectively. This suggests that 60 minutes after the GnRH agonist stimulation test was adequate to distinguish the pubertal from the prepubertal state and only taking samples at 0, 30, and 60 minutes after the GnRH analogue stimulation test could effectively reduce the number of blood samples required while still achieving good diagnostic accuracy. The other LH/FSH time points also showed extremely high sensitivity and specificity, but the 60-minute post-stimulus gonadotropin is a better choice considering the additional time that would be needed.

Previous studies have generally paid more attention to the peak LH value, ignoring the LH/FSH ratio. The present study found that the LH/FSH ratio was more efficient for the diagnosis of children with secondary sexual characteristics. Children with a high LH/FSH ratio also generally have a high LH peak. It is important to note, however, that an abnormal LH level is not sufficient to make a diagnosis, which must be based on the presence of clinical manifestations of secondary sexual development. Besides, it’s our study limitation that using any numerical cut off for stimulation testing, will falsely categorize some patients as having pubertal activation of the pituitary-gonadal axis. Hence it is extremely important to verify pubertal progression especially in girls. In addition, this study is a retrospective study, and the data of target height-SDS is limited, which will be further supplemented and verified in future prospective studies.

## Conclusion

Basal LH and LH/FSH measurement can be used to identify female patients with CPP. When the baseline value of LH is > 0.535 mIU/L, the diagnosis of CPP can be confirmed without a GnRH agonist stimulation test. A single 60-minute post-stimulus gonadotropin result of LH and LH/FSH can be used instead of a GnRH agonist stimulation test, or samples can be taken only at 0, 30, and 60 minutes after a GnRH agonist stimulation test. This reduces the number of blood draws required compared with the traditional stimulation test, while still achieving a high level of diagnostic accuracy.

## Data Availability Statement

The original contributions presented in the study are included in the article/supplementary material. Further inquiries can be directed to the corresponding author.

## Ethics Statement

The studies involving human participants were reviewed and approved by Ethics Committee of the Second Affiliated Hospital and Yuying Children’s Hospital of Wenzhou Medical University. Written informed consent to participate in this study was provided by the participants’ legal guardian/next of kin.

## Author Contributions

RC and PL conceived and designed research. RC, PF, and JL conducted experiments and analyzed data. PF, ZL and YZ wrote the manuscript. All authors contributed to the article and approved the submitted version.

## Conflict of Interest

The authors declare that the research was conducted in the absence of any commercial or financial relationships that could be construed as a potential conflict of interest.

## Publisher’s Note

All claims expressed in this article are solely those of the authors and do not necessarily represent those of their affiliated organizations, or those of the publisher, the editors and the reviewers. Any product that may be evaluated in this article, or claim that may be made by its manufacturer, is not guaranteed or endorsed by the publisher.
